# Low Distribution of TIM-3^+^ Cytotoxic Tumor-Infiltrating Lymphocytes Predicts Poor Outcomes in Gastrointestinal Stromal Tumors

**DOI:** 10.1155/2021/6647292

**Published:** 2021-02-17

**Authors:** Chun Zhuang, Bo Ni, Zi-Zhen Zhang, Wen-Yi Zhao, Lin Tu, Xin-Li Ma, Lin-Xi Yang, Hui Cao, Ming Wang

**Affiliations:** Department of Gastrointestinal Surgery, Renji Hospital, Shanghai Jiao Tong University School of Medicine, Shanghai, China

## Abstract

There are multiple tumor-infiltrating lymphocytes (TILs) and relevant immune checkpoints existing in gastrointestinal stromal tumor (GIST), which provides opportunities and rationales for developing effective immunotherapies. Recent studies have suggested that checkpoint TIM-3/Gal-9 plays a pivotal role on immune response in multiple tumors, similar to the PD-1/PD-L1, emerging as a potential therapeutic target. However, their functions in GIST are unrevealed. Hence, the expression of immune checkpoints TIM-3 and Gal-9, as well as the infiltration of CD8^+^ T cells and NK cells, is described in 299 cases of GIST specimens. The results showed that TIM-3 and Gal-9 are mainly expressed in TILs, rarely in tumor cells. Expression levels of TIM-3 and Gal-9 significantly differ in varying risks of GIST and exert opposite distribution trends. Indicated by prognosis analysis, high TIM-3 expression of TILs was associated with improved outcome, while low expression levels of TIM-3 in combination with low amounts of CD8^+^ and CD56^+^ TILs predict extremely poor survival. The integrated analysis of TIM-3^+^, CD8^+^, and CD56^+^ TILs as one biomarker is a reliable independent predictor of prognosis. In conclusion, low densities of TIM-3^+^ TILs are associated with poor survival, and integrated immune biomarkers lead to superior predictors of GIST prognosis.

## 1. Introduction

GIST is the most common mesenchymal neoplasm of the gastrointestinal (GI) tract, most commonly found in the stomach (60%) and proximal portions of the small intestine (30%), even generating from any parts of the GI tract [1]. Somatic gain-of-function mutations in *c-KIT* are the most common genetic driver aberrations, which account for approximately 80% of all GISTs [2]. In addition to surgery, imatinib mesylate, a tyrosine kinase inhibitor, is the most effective treatment of irresectable and metastatic GIST patients, whose clinical response achieves up to 80% of all cases [3, 4]. However, drug resistance occurs frequently due to secondary mutations of *c-KIT*. Moreover, ~14% of GISTs have *de novo* or primary resistance to the imatinib [5, 6].

Many investigators have shown that abundant tumor-infiltrating lymphocytes (TILs) are located in the microenvironment of GISTs, playing an important role in tumor surveillance and progression and enhancing the antitumor function of imatinib [7, 8]. Immunotherapeutic strategies, utilizing different components of immune system to eliminate viable tumor cells, are a promising therapeutic strategy of GIST. The presence of TILs in GIST and their apparent roles are intriguing and provide rationales for the development of immunotherapy.

T cell immunoglobulin-3 (TIM-3) and its ligand galectin-9 (Gal-9), the same as programmed cell death protein-1 (PD-1) and its ligands (PD-L1/PD-L2), were considered key immune checkpoint inhibitors driving the immune escape [7, 9]. TIM-3, as a cell surface receptor differentially expressed on mature T lymphocytes, was found to be expressed on Th1 (IFN-*γ*-producing CD4^+^ T helper 1) cells and Tc1 (CD8^+^ T cytotoxic 1) cells, but not on Th2 cells [10]. And this means that induction of TIM-3/Gal-9 inhibitory signaling pathways during CD8^+^ T cells' response to tumor cells results in CD8^+^ T cell exhaustion that drives tumor progression [11]. TIM-3 often coexpresses with PD-1 in tumor-infiltrating CD8^+^ T cells. And in many cancer researches, the antitumor effect of coblockade of the TIM-3 and PD-1 pathways is superior to blocking PD-1 alone [12, 13]. As known, the therapeutic effects of immune checkpoint antibodies depend on the expression levels of target molecules. No studies have systematically examined the expression patterns alone or coexpression of multiple immune inhibitory molecules in GIST specimens before. And making their expression patterns clear in GIST could establish reliable targets of these molecules, or combinations, promising for clinical therapy. Thus, the primary aim of this study was to examine the distribution of different TILs, including several immune inhibitory molecules in GIST tissue, explore their correlations, and reveal their effects on cancer-related survival. TIM-3, PD-1, PD-L1, CD8, and CD56 were chosen for study due to their widely known roles on tumor immune; meanwhile, the reliable primary antibodies are available.

## 2. Materials and Methods

### 2.1. Antibodies

The primary antibodies involved were as follows: CD8 (CST, #85336, 1 : 200), CD56 (CST, #3576, 1 : 200), TIM-3 (Abcam, ab185703, 1 : 200), and Gal-9 (Abcam, ab69630, 1 : 200).

### 2.2. Tumor Specimens

Archived formalin-fixed paraffin-embedded tumor tissue from 299 GIST patients who were pathologically diagnosed as GIST and treated at Renji Hospital, from September 2004 to September 2013, was used for tissue microarray (TMA) construction in this study. These patients did not receive any adjuvant treatment during the perioperative periods. For TMA construction, specimens from GIST patients were firstly stained by hematoxylin-eosin to identify the tumor region, avoiding the normal tissue or necrosis sites, followed by selecting a 1.5 mm-diameter tissue as a core. Clinical information was retrospectively collected from the hospitalization archives, as shown in Table 1. Complete follow-up data for GIST patients in cohort were also available. The study was approved by the Research Ethics Committee of Renji Hospital. In addition, the study protocol conforms to the ethical guidelines of the 1975 Declaration of Helsinki. All the patients involved in this study had signed informed consent. Ethical approval number was 2018029.

### 2.3. Immunohistochemistry (IHC)

IHC for CD8, CD56, TIM-3, and Gal-9 was performed according to previous description [14]. Tumor-infiltrating immune cells were classified semiquantitatively, which was independently scored by two pathologists blinded to clinical outcomes and differences resolved by mutual agreement, as previously described [15]. The stained TMA was observed in ×200 magnification and selected randomly for five views to evaluate the intensity of immune infiltration in the GIST. The intensity of immune infiltration was assigned a semiquantitative score as “-,” “+,” “++,” and “+++”: “-“ = “none” (no staining), “+” = “few scattered infiltrating immune cells,” “++” = “moderate infiltrating immune cells,” or “+++” = “ dense infiltrating immune cells.” And “++” and “+++” were considered high immune marker groups; the others were considered low immune marker groups.

### 2.4. Statistical Analysis

The infiltrating immune cells and tumor characteristics were evaluated using the Fisher exact test and Chi-square test in SPSS 20. Correlation analysis was obtained using the Spearman coefficient method. The Kaplan Meier (KM) plots for both single and integrated markers were performed for survival analysis via the log-rank test or Cox regression analysis. Statistical significance and hazard ratio along with 95% confidence intervals for the KM plots were established using the log rank *P* value, obtained from Cox proportional hazard regression analysis. All tests were two-sided except as indicated, and *P* values of <0.05 were considered significant.

## 3. Results

### 3.1. Baseline Clinicopathologic Characteristics of GIST Patients in our Study

Our study involved a total of 299 GIST patients, who were pathologically diagnosed from September 2004 to September 2013 and did not receive any postoperative adjuvant therapy for various reasons. Their characteristics are summarized in Table 1. The median age was 59 years, and 53.8% were male. The mean tumor size was 7.2 cm and median 6.0 cm, with range 0.5–30 cm. Regarding the modified National Institute of Health (NIH) consensus, 39.1% of samples were predicted as low risk, 15.7% as intermediate risk, and 45.2% as high risk, of which 58 (19.4%) cases occur with local invasion. The most frequent anatomical site was the stomach (55.9%), followed by the small intestine (32.1%). More than half of the patients (66.2%) had mitotic counts of less than 5 per 50 high-power fields (HPFs). Tumor bleeding was present in 53 of 299 samples (17.7%). In this cohort, complete follow-up data of GIST patients were available. Sixty-three patients (21.1%) developed into relapses during follow-up. The median overall survival (OS) and median disease-free survival (DFS) were 56 months and 53 months, respectively.

### 3.2. Distribution of TIM-3^+^, Gal-9^+^, CD8^+^, and CD56^+^ TILs in GISTs

**Figure 1:**
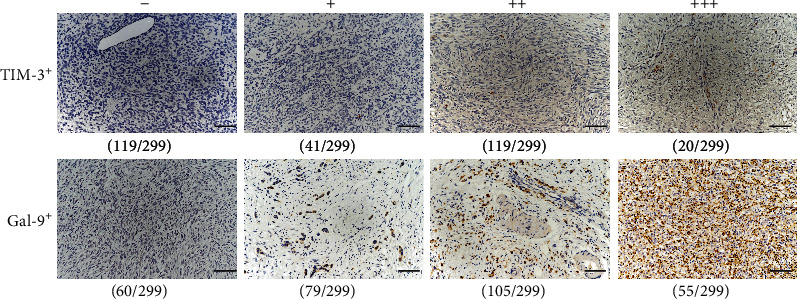
Representative images of individual expression levels of TIM-3 and Gal-9 are shown with their corresponding frequencies in GIST cohorts. The symbol in the top indicates the intensity scores of indicated immune markers. The number in the bottom of every picture indicates the corresponding amounts of the score (original magnification: ×200; Scale bars, 200 *μ*m).

Uncovered by IHC in Figure 1, we found that rare cytoplasmic or membrane localization of TIM-3/Gal-9 was observed in tumor cells, whereas predominantly located in the infiltrating lymphocytes [16]. GISTs are the most common mesenchymal neoplasms of the gastrointestinal tract and all the lymphocytes counted in one field of a tissue point in the TMA were analyzed as a whole. Infiltrating levels of lymphocytic cells were described ranging from none, scattered, moderate, to dense infiltrates, which scored as “-,” “+,” “++,” and “+++,” respectively. A detailed list of the densities of TIM-3^+^, Gal-9^+^, CD8^+^, and CD56^+^ TILs and representative images are provided in Figure 1 and Figure S1. The staining intensities for TIM-3 were “++” of 39.8% (119/299) and “+++” of 6.7% (20/299) among all cases, defined as positive groups. As for Gal-9, the positive cases were identified to be 150 patients, which were scored as “++” (35.1%, 105/299) and “+++” (18.4%, 55/299). A detailed description of CD8^+^ and CD56^+^ TILs in GIST was shown in Figure S1.

### 3.3. Association of TIM-3, Gal-9, CD8, and CD56 Distribution with Histologic Features

Previous studies have reported that TILs populate in the microenvironment of GISTs. These cells have an important role in tumor surveillance and progression [7]. To address this issue in GISTs, we examined the relationship between the expression of tumor-infiltrating T-cell subset density and the clinicopathological of GIST patients (Table 2). The Chi-square test revealed that TIM-3 and Gal-9 were significantly correlated with NIH risk (*P* < 0.001 and *P* < 0.001), tumor size (*P* < 0.001 and *P* = 0.009), mitotic count (*P* < 0.001 and *P* = 0.035), and recurrence (*P* = 0.038 and *P* < 0.001). In addition, Gal-9 expression was significantly correlated with ki67 classification (*P* = 0.030) and local invasion (*P* = 0.041). CD8 T cells and CD56 NK cells, frequently immune cells found in GIST, were significantly correlated with NIH risk (*P* < 0.001 and *P* = 0.001), tumor size (*P* = 0.004 and *P* = 0.004), mitotic count (*P* = 0.001 and *P* = 0.004), and local invasion (*P* = 0.028 and *P* = 0.015).

### 3.4. Correlation between Densities of TIL Subsets and Survival of GIST Patients

**Figure 2:**
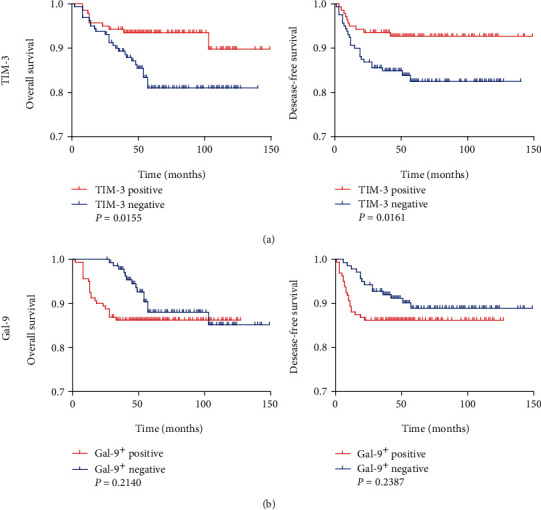
The Kaplan-Meier plots of overall and disease-free survival grouped by TIM-3^+^ (a) and Gal-9^+^ (b) TIL densities in GIST. Optimal positive vs. negative values were established by scoring the IHC staining. *P* values were determined by the log-rank test.

Next, we investigated the relationship between density of TIL subsets and prognosis of patients in the collected cohort. Optimal cut-offs for negative versus positive staining were made as described above. We found that no or low expression of TIM-3 (Figure 2(a), *P* = 0.0155), CD8 (Figure S2(a), *P* = 0.0047), and CD56 (Figure S2(b), *P* = 0.0367) were associated with poor GIST-specific survival, while expression of Gal-9 (Figure 2(b), *P* = 0.2140) was not associated with GIST-specific survival. For GIST progression, the relationship between prognosis with expression levels of these markers followed the same trends. Low expression of TIM-3 (Figure 2(a), *P* = 0.0161), CD8 (Figure S2(a), *P* = 0.0050), and CD56 (Figure S2(b), *P* = 0.0480) were significantly associated with shorter time to GIST recurrence, while Gal-9 (Figure 2(b), *P* = 0.2140) was not associated with GIST recurrence. Furthermore, univariate and multivariate Cox regression analyses showed that TIM-3 levels were an independent predictor of GIST patients' overall survival and disease-free survival (Table S1).

### 3.5. Integrated Analyses of Multiple TIL Subsets on Survival of Patients

**Figure 3:**
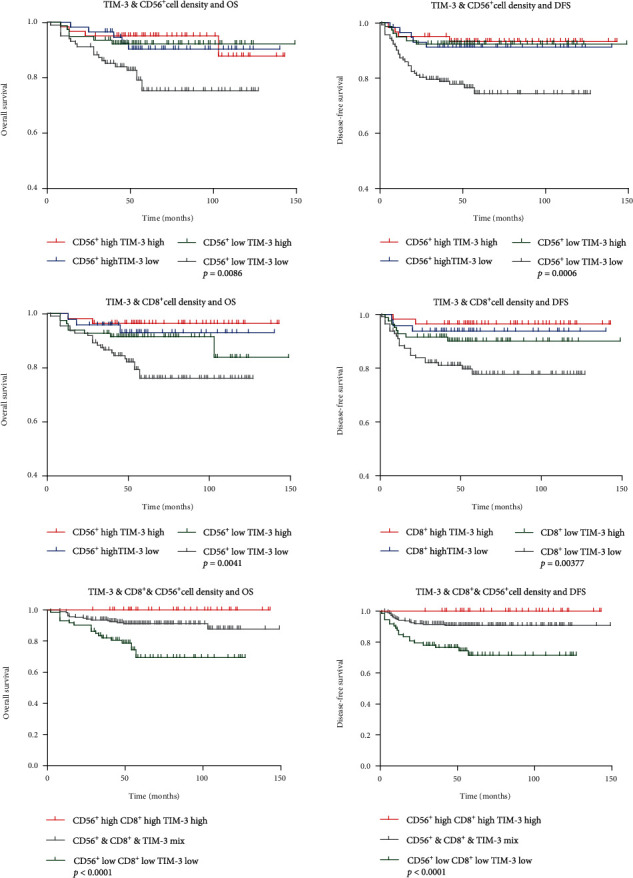
The Kaplan-Meier plots of overall (left column) and disease-free (right column) survival grouped by the integrated TIM-3^+^, CD8^+^, and CD56^+^ TIL amount. Low levels of two or three of these TIL markers predicted extremely poor prognosis.

Owing to the potentials of the TIL subset density to predict prognosis of GIST, we wondered whether combining two or three of the independent prognostic immune parameters (TIM-3, CD8, and CD56) would improve the prediction efficiency. Integrated analysis showed that low levels of two or three of these TIL parameters predicted worse prognosis compared to other combinations, as shown in Figure 3. Concordantly, like NIH risk criteria, the combined consideration of TIM-3^+^, CD8^+^, and CD56^+^ TIL as one biomarker was a powerful independent predictor of prognosis in multivariate analysis (*P* < 0.001, HR = 0.397, 95%CI = 0.206–0.766).

### 3.6. Distribution Correlation between Various TIL Subsets in GIST

**Figure 4:**
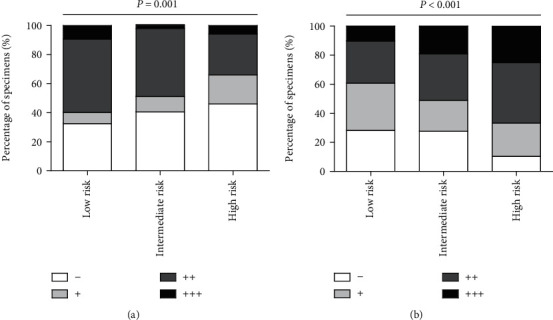
Statistical analysis of IHC scores for TIM-3^+^ (a) and Gal-9^+^ (b) TIL distribution in different risk of GIST patients, *P* = 0.001 and *P* < 0.001, respectively.

**Figure 5:**
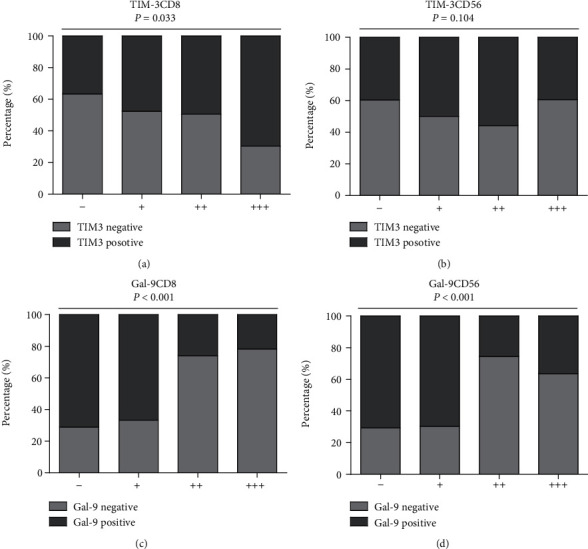
Distribution correlation among various TIL subsets. (a) The higher the densities of CD8^+^ T cells, the more TIM-3 expression was presented. (b) No significant correlation was presented between TIM-3 expression and CD56 distribution. (c, d) The lower the degrees of CD8 (c) and CD56 (d), the more Gal-9 expression was presented.

In the correlation analysis of TIL subsets' distribution with histologic features (Table 2), we found distinct distribution of TIM-3 and Gal-9 with individual pathological parameters. For instance, TIM-3 expression was higher in low-risk samples than high-risk samples (Figure 4(a), *P* = 0.001), while higher Gal-9 expression existed in high-risk samples (Figure 4(b), *P* < 0.001), hinting antagonistic prognostic effects of TIM-3 and Gal-9. To further estimate whether various infiltrating types collaborated in improving the clinical course of GIST, Spearman's correlation coefficients were calculated among different TIL subsets (Table 3). The results showed that TIM-3 expression was strongly positively associated with the density of CD8^+^ T cells (*r* = 0.182, *P* = 0.002 and *r* = 0.127, *P* = 0.008, respectively). And the Chi-square test showed that the higher densities of CD8^+^ T cells, the more TIM-3 expression was presented (*P* = 0.033, Table 4 and Figure 5(a)), while negative association existed between Gal-9 expression and CD8^+^ T cells (*r* = −0.316, *P* < 0.001), as well as CD56^+^ NK cells (*r* = −0.326, *P* < 0.001). And the Chi-square test showed that the lower densities of CD8^+^ and CD56^+^ cells, the more Gal-9 expression were presented (*P* < 0.001, Table 4 and Figures 5(c) and 5(d)). All the evidences reflected a potentially immunosuppressive microenvironment in GIST, indicating that different immunosuppressive effects were based on different types of TILs.

## 4. Discussion

The aim of this study was to evaluate the expression levels of TIM-3 and its ligands Gal-9 in GIST and to link the expression patterns with clinical prognosis parameters. From the IHC results, we found that predominantly staining of indicated immune checkpoint molecules was located on lymphocytes, whereas rare cytoplasm or membrane distribution was observed in tumor cells. This phenomenon means that GIST escapes from immune surveillance by inactivating immune responses [17]. In our study, firstly conducted in a GIST cohort, we showed that expression of immune checkpoints differs according to tumor biology and patient characteristics. Patients harboring low risk, small size, and low mitoses had highly abundant TIM-3^+^ TILs, whereas opposite phenomena were presented with Gal-9. Besides, the relationship among TIL subsets showed positive correlation between TIM-3^+^ and CD8^+^ TIL and negative correlation between Gal-9^+^ and other types. These conflicting evidences are probably due to different immune-escaped mechanism by TIM-3 and Gal-9, which needed to further studied. What is more, no significant correlations were presented between TIM-3 expression and CD56 distribution (Figure 5(b)). As the scores “-,” “+,” and “++” of CD56 representing the increasing densities of NK cells, higher expression of TIM-3 emerged. More patients were required here to assess the relationship between TIM-3 expression and NK cells distribution.

Subsequently, in the analysis of the relationship between different TIL subsets and patients' survival, we found that low TIM-3^+^, as same as low CD8^+^ and CD56^+^ TILs, predicted poor survival and short time to relapse, respectively. Further analysis showed that the combination of TIM-3^+^, CD8^+^, and CD56^+^ TIL densities appeared to be a powerful independent predictor of GIST-specific survival. We demonstrated that there was a group of patients with extremely poor prognosis who owned low densities of TIM-3^+^, CD8^+^, and CD56^+^ TILs. One may wonder why low expression of these molecules signifies high risk of GIST, especially the immune checkpoint-inhibiting molecules. It is known that Gal-9 and PD-L1 are widely expressed in multiple cell types, and their expressions are overexpressed in response to IFN-*γ* and TIL activation, a process called adaptive immune resistance [15, 18, 19]. For homeostasis, highly expressed Gal-9 and PD-L1 perform immunosuppressive effects. Probably, low or no expression of such molecules indicates that the cancer is beyond detection by the immune system or that the antitumor immune response is inactivated.

As known, TIM-3 mainly located on the cytomembrane and its expression in T cells modulated immune response [20]. Previously known, increased TIM-3 expression in monocytes/macrophages [21], peripheral NK cells [22], and tumor infiltrating T cells [18, 23, 24] would lead to poor prognosis in various cancers. However, in our study, low expression of TIM-3 was found to be associated with worse outcome, which was similar with that in prostate cancer [25]. The mechanism that causes this difference may be that TIM-3 expressed in tumor-associated immune cells had different tumorigenic patterns in different tumor microenvironments [26, 27]. Moreover, unlike other tumors, rare staining in tumor cells was detected in GIST, owing its derivation from mesenchymal cells [28, 29]. This phenomenon indicates that different localization of TIM-3 exerts totally different functions in tumor biology. These conflicting phenomena may also result from the suppressive immune microenvironments in GIST, where the cytotoxic immune cells have not been activated with little expression of checkpoints.

Rare studies have shown the association of TIM-3 and TILs in GIST, and our study firstly describes this relationship in GIST. According to the model by Teng et al. [30], we try to stratify the risks of GIST based on the TIM-3 expression and the presence or absence of TILs. Immune silence occurs in patients with low infiltration of TIM-3^+^ and CD8^+^ TILs (type II tumors), exerting the worst prognosis, whereas patients with high TIM-3 expression and abundant TILs (type I tumors) have the best prognosis as predicted due to the activating immune response. Moreover, our data demonstrates that GISTs with an ongoing antitumor immune response (high TIM-3 and high TIL densities) are the candidates to benefit from TIM-3 blockade. Nevertheless, more clinical trials need to be conducted to verify the suggestion above.

In conclusion, this study shows that TIM-3 is predominantly expressed in TILs of resected GISTs. Low expression of TIM-3 in TILs reflects an aggressive tumor biology with impaired OS, and combined with low densities of CD8^+^ and CD56^+^, TILs predict extremely poor survival. TIL-expressing TIM-3 should be induced in response to immunologic pressure, which explains why their presence is associated with prolonged survival and emerges as a pivotal marker to guide immunotherapeutic candidates of GIST.

## Figures and Tables

**Table 1:** Clinicopathological characteristics of GIST patients (*N* = 299).

Characteristics		*N* (%)
Age (years)	≤59	162 (54.2%)
	>59	137 (45.8%)
Gender	Male	161 (53.8%)
	Female	138 (46.2%)
Modified NIH criteria	Low risk	117 (39.1%)
	Intermediate risk	47 (15.7%)
	High risk	135 (45.2%)
Tumor size	≤5 cm	133 (44.5%)
	>5 cm	166 (55.5%)
Tumor site	Stomach	167 (55.9%)
	Small bowel	96 (32.1%)
	Colon	7 (2.3%)
	Others	29 (9.7%)
Mitoses per 50 HPFs	≤5	198 (66.2%)
	5-10	44 (14.7%)
	≥10	57 (19.1%)
Tumor bleeding	Yes	53 (17.7%)
	No	246 (82.3%)
Ki67 classification	I	204 (68.2%)
	II	43 (14.4%)
	III	52 (17.4%)
Recurrence state	Yes	63 (21.1%)
	No	236 (78.9%)
Local invasion	Yes	58 (19.4%)
	No	241 (80.6%)

Abbreviations: HPF, high power field.

**Table 2:** Correlation of TIM-3^+^, Gal-9^+^, CD8^+^, and CD56^+^ TIL densities with clinicopathological characteristics.

Characteristics	TIM-3^+^		*P* value	Gal-9^+^		*P* value	CD8^+^		*P* value	CD56^+^		*P* value
	Low	High		Low	High		Low	High		Low	High	
Age (years)			0.441			0.873			**0.035**			0.283
≤59	90	72		76	86		97	65		93	69	
>59	70	67		63	74		98	39		87	50	
Gender			0.668			0.508			0.465			0.827
Male	88	73		72	89		108	53		96	65	
Female	72	66		67	71		87	51		84	54	
Modified NIH criteria			**<0.0001**			**<0.0001**			**<0.0001**			**0.001**
Low risk	47	70		71	46		60	57		55	62	
Intermediate risk	24	23		23	24		35	12		31	16	
High risk	89	46		45	90		100	35		94	41	
Tumor size			**<0.0001**			**0.009**			**0.004**			**0.004**
≤5 cm	56	77		73	60		75	58		68	65	
>5 cm	104	62		66	100		120	46		112	54	
Tumor site			0.419			0.088			**<0.0001**			0.153
Stomach	93	74		76	91		124	43		108	59	
Small bowel	45	51		52	44		42	54		50	46	
Colon	4	3		3	4		5	2		3	4	
Others	18	11		8	21		24	5		19	10	
Mitoses per 50 HPFs			**<0.0001**			**0.035**			**0.001**			**0.004**
≤5	92	106		102	96		115	83		108	90	
5-10	24	20		14	30		32	12		27	17	
≥10	44	13		23	34		48	9		45	12	
Tumor bleeding			0.679			0.913			0.857			0.339
Yes	27	26		25	28		34	19		35	18	
No	133	113		114	132		161	85		145	101	
Ki67 classification			0.099			**0.030**			0.357			0.275
I	101	103		99	105		128	76		117	87	
II	25	18		24	19		29	14		27	16	
III	34	18		16	36		38	14		36	16	
Recurrence state			**0.038**			**<0.0001**			**<0.0001**			0.078
Yes	41	22		16	47		55	8		44	19	
No	119	117		123	113		140	96		136	100	
Local invasion			0.080			**0.041**			**0.028**			**0.016**
Yes	37	21		20	38		45	13		43	15	
No	123	118		119	122		150	91		137	104	

Abbreviations: HPF, high power field. Note: The values in bold type are those with statistical significance (*P* < 0.05).

**Table 3:** Distribution correlation of various TIL subsets in GIST.

	CD8^+^ cell	CD56^+^ cell	TIM-3^+^ cell	Gal-9^+^ cell
CD8^+^ cell		*r* = 0.244 *P* < 0.001	*r* = 0.182 *P* = 0.002	*r* = −0.316 *P* < 0.001
CD56^+^ cell	*r* = 0.244 *P* < 0.001		*r* = 0.106 *P* = 0.068	*r* = −0.326 *P* < 0.001
TIM-3^+^ cell	*r* = 0.182 *P* = 0.002	*r* = 0.106 *P* = 0.068		*r* = −0.073 *P* = 0.207
Gal-9^+^ cell	*r* = −0.316 *P* < 0.001	*r* = −0.326 *P* < 0.001	*r* = −0.073 *P* = 0.207	

Note: Spearman's correlation coefficients were calculated with two-sided models.

**Table 4:** Association of TIM-3, Gal-9 levels with CD8^+^ and CD56^+^ TIL densities in GIST.

TIL density	TIM-3		*P* value	Gal-9		*P* value
	Low	High		Low	High	
CD8^+^ cell			0.033			*P* < 0.0001
-	57	33		26	64	
+	55	50		35	70	
++	41	40		60	21	
+++	7	16		18	5	
CD56^+^ cell			0.104			*P* < 0.0001
-	70	46		34	82	
+	32	32		20	44	
++	38	48		64	22	
+++	20	13		21	12	

Note: Chi-square tests were conducted for *P* values.

## Data Availability

The data used to support the findings of this study are available from the corresponding author upon request.

## Supplementary Materials

Supplementary 1 Representative images for expression levels of CD8 and CD56 were shown with their frequencies in GIST cohorts. The symbol in the top indicated the density scores of indicated immune markers. The number in the bottom of every image indicated the corresponding amounts of the score (original magnification: ×200; scale bars: 200 *μ*m).

Supplementary 2 The Kaplan-Meier plots of overall (left column) and disease-free (right column) survival grouped by CD8^+^ (a) and CD56^+^ (b) TIL densities in GIST. Optimal high vs. low density was established by scoring the IHC staining. *P* values were determined by log-rank test.

Supplementary 3 The concise information of supplementary tables was described before each table.
