# Supplementary figures and images for: Low Distribution of TIM-3+ Cytotoxic Tumor-Infiltrating Lymphocytes Predicts Poor Outcomes in Gastrointestinal Stromal Tumors
Source: J Immunol Res. 2021 Feb 17;2021:6647292. doi: 10.1155/2021/6647292 (PMC7907748; doi:10.1155/2021/6647292)

Supplementary Figure 1

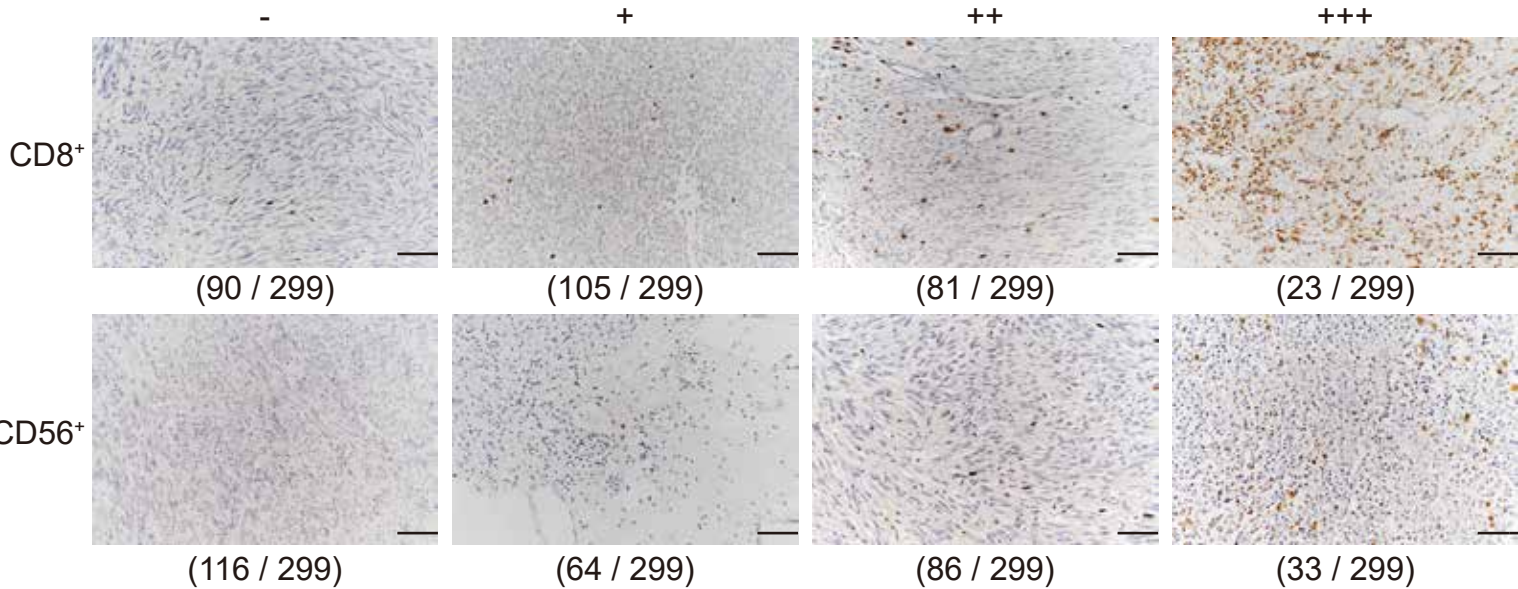

Supplement: Supplementary 1 — Representative images for expression levels of CD8 and CD56 were shown with their frequencies in GIST cohorts. The symbol in the top indicated the density scores of indicated immune markers. The number in the bottom of every image indicated the corresponding amounts of the score (original magnification: ×200; scale bars: 200 μm). [file 6647292.f1.pdf]

Supplementary Figure 2

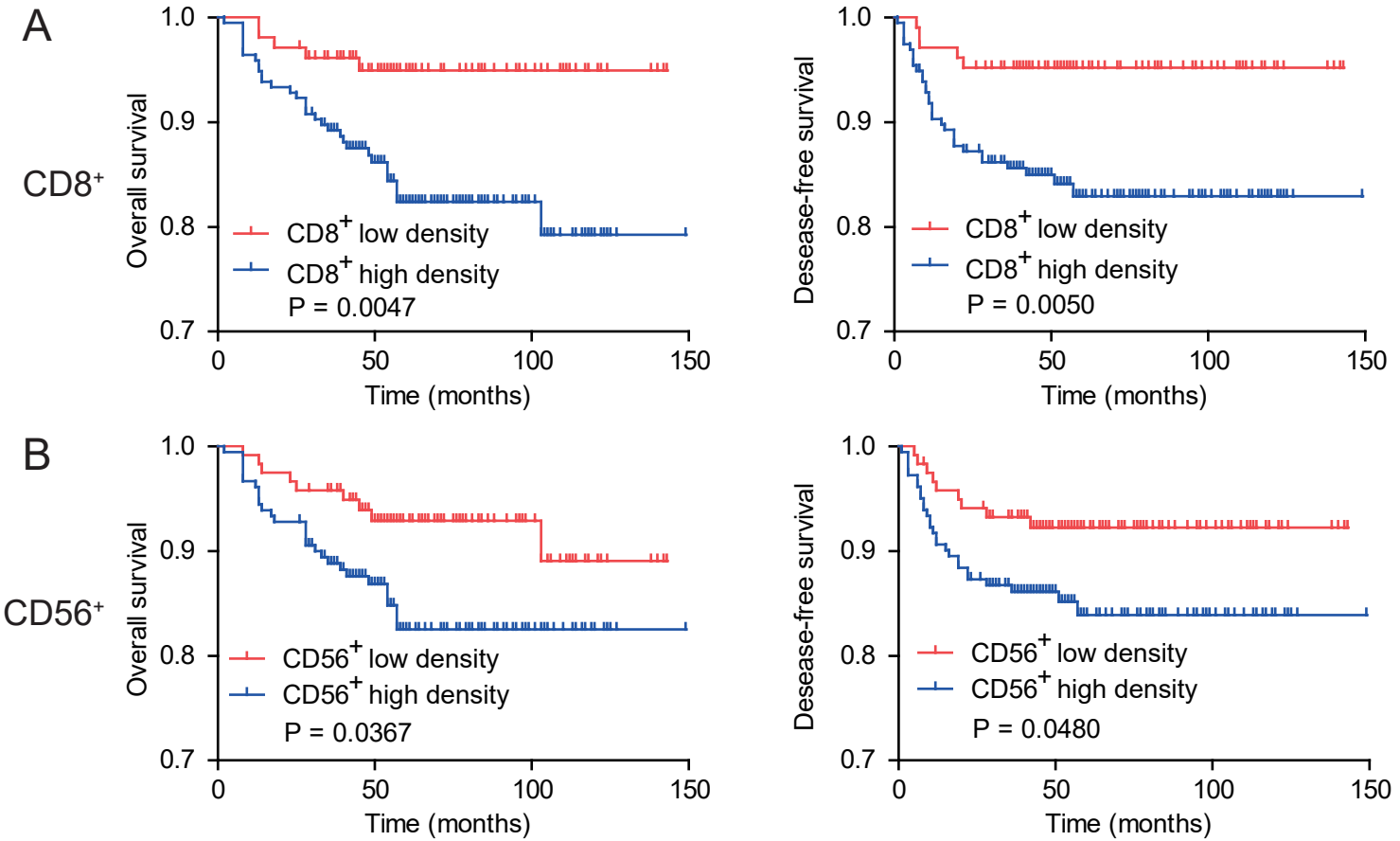

Supplement: Supplementary 2 — The Kaplan-Meier plots of overall (left column) and disease-free (right column) survival grouped by CD8+ (a) and CD56+ (b) TIL densities in GIST. Optimal high vs. low density was established by scoring the IHC staining. P values were determined by log-rank test. [file 6647292.f2.pdf]
