# Supplementary material for: Low Distribution of TIM-3+ Cytotoxic Tumor-Infiltrating Lymphocytes Predicts Poor Outcomes in Gastrointestinal Stromal Tumors
Source: J Immunol Res. 2021 Feb 17;2021:6647292. doi: 10.1155/2021/6647292 (PMC7907748; doi:10.1155/2021/6647292)
Supplement: Supplementary 3 — The concise information of supplementary tables was described before each table. [file 6647292.f3.docx]

**Supplementary** **Tables**

**Supplementary Table 1.** Univariate and Multivariate Cox Regression Analysis of Prognostic Factors on Overall Survival in GIST.

| Factors | Univariate | | Multivariate | |
| --- | --- | --- | --- | --- |
|  | HR (95% CI) | P value | HR (95% CI) | P value |
| Age | 1.010  (0.982 to 1.038) | 0.491 |  |  |
| Gender  (male vs. female) | 1.746  (0.873 to 3.492) | 0.115 |  |  |
| Location  (stomach vs. non-stomach) | 0.517  (0.266 to 1.003) | 0.051 | 0.771  (0.390 to 1.524) | 0.455 |
| Risk stratification  (high vs non-high risk) | 16.024  (4.912 to 52.271) | **<0.0001** | 9.174  (2.528 to 33.794) | **0.001** |
| Local invasion  (present vs. absent) | 9.680  (4.885 to 19.179) | **<0.0001** | 4.001  (1.921 to 8.337) | **<0.0001** |
| TIM-3 expression  (low vs. high expression) | 2.219  (1.070 to 4.602) | **0.032** | 2.166  (1.044 to 4.497) | **0.038** |

Note: The values in bold type are those with statistical significance (P < 0.05).

**Supplementary Table 2.** Univariate and Multivariate Cox Regression Analysis of Prognostic Factors on Disease-Free Survival in GIST.

| Factors | Univariate | | Multivariate | |
| --- | --- | --- | --- | --- |
|  | HR (95% CI) | P value | HR (95% CI) | P value |
| Age | 1.008  (0.987 to 1.029) | 0.451 |  |  |
| Gender  (male vs. female) | 1.731  (1.031 to 2.906) | **0.038** | 1.417  (0.836 to 2.400) | 0.195 |
| Location  (stomach vs. non-stomach) | 0.422  (0.253 to 0.701) | **0.001** | 0.986  (0.582 to 1.670) | 0.958 |
| Risk stratification  (high vs non-high risk) | 16.138  (6.948 to 37.486) | **<0.0001** | 10.180  (4.073 to 25.445) | **<0.0001** |
| Local invasion  (present vs. absent) | 8.675  (5.232 to 14.383) | **<0.0001** | 3.227  (1.885 to 5.523) | **<0.0001** |
| TIM-3 expression  (low vs. high expression) | 2.720  (1.542 to 4.797) | **0.001** | 3.052  (1.724 to 5.404) | **<0.0001** |

Note: The values in bold type are those with statistical significance (P < 0.05).
